# Beyond resorption: osteoclasts as drivers of bone formation

**DOI:** 10.1186/s13619-024-00205-x

**Published:** 2024-10-11

**Authors:** Qianfeng Xiang, Lei Li, Wei Ji, Debby Gawlitta, X Frank Walboomers, Jeroen J.J.P. van den Beucken

**Affiliations:** 1 Radboudumc, Dentistry – Regenerative Biomaterials, Philips Van Leijdenlaan 25, Nijmegen, 6525EX the Netherlands; 2 State Key Laboratory of Oral & Maxillofacial Reconstruction and Regeneration, Key Laboratory of Oral Biomedicine Ministry of Education, Hubei Key Laboratory of Stomatology, School & Hospital of Stomatology, Wuhan University, Wuhan, China; 3 Department of Oral and Maxillofacial Surgery & Special Dental Care, University Medical Center Utrecht, Utrecht University, Utrecht, GA 3508 The Netherlands; 4 Regenerative Medicine Center Utrecht, Utrecht, CT 3584 The Netherlands; 5 Research Institute for Medical Innovation, Radboudumc, Nijmegen, the Netherlands

**Keywords:** Osteoclast, Osteoclatogenesis, Osteoclast characterization, Angiogenesis regulation, Bone formation, Bone regeneration

## Abstract

Emerging evidence illustrates that osteoclasts (OCs) play diverse roles beyond bone resorption, contributing significantly to bone formation and regeneration. Despite this, OCs remain mysterious cells, with aspects of their lifespan—from origin, fusion, alterations in cellular characteristics, to functions—remaining incompletely understood. Recent studies have identified that embryonic osteoclastogenesis is primarily driven by osteoclast precursors (OCPs) derived from erythromyeloid progenitors (EMPs). These precursor cells subsequently fuse into OCs essential for normal bone development and repair. Postnatally, hematopoietic stem cells (HSCs) become the primary source of OCs, gradually replacing EMP-derived OCs and assuming functional roles in adulthood. The absence of OCs during bone development results in bone structure malformation, including abnormal bone marrow cavity formation and shorter long bones. Additionally, OCs are reported to have intimate interactions with blood vessels, influencing bone formation and repair through angiogenesis regulation. Upon biomaterial implantation, activation of the innate immune system ensues immediately. OCs, originating from macrophages, closely interact with the immune system. Furthermore, evidence from material-induced bone formation events suggests that OCs are pivotal in these de novo bone formation processes. Nevertheless, achieving a pure OC culture remains challenging, and interpreting OC functions in vivo faces difficulties due to the presence of other multinucleated cells around bone-forming biomaterials. We here describe the fusion characteristics of OCPs and summarize reliable markers and morphological changes in OCs during their fusion process, providing guidance for researchers in identifying OCs both in vitro and in vivo. This review focuses on OC formation, characterization, and the roles of OCs beyond resorption in various bone pathophysiological processes. Finally, therapeutic strategies targeting OCs are discussed.

## Background

Osteoclasts (OCs) are multinucleated cells that play a pivotal role in maintaining bone homeostasis (Hattner et al. 1965). Traditionally, OCs have been regarded as monofunctional cells with the mere purpose of bone resorption. However, an emerging body of evidence has unveiled additional functionality of OCs, in bone tissue also contributing toward anabolic physiological processes (Faqeer et al. 2023; Hattner et al. 1965; Lotinun et al. 2013; Oursler 1994; Xian et al. 2012; Xie et al. 2014). These notable discoveries have attracted interest among scientists, leading to a paradigm shift in the investigation on the role of OCs in bone formation, regeneration and their potential applications.

During different stages of development, OCs arise from distinct sources. In the embryonic period, EMP-derived OCs predominate, but are gradually replaced by HSC-derived osteoclast precursors (OCPs) through a heterogeneous fusion process (Jacome-Galarza et al. 2019; Yahara et al. 2020). This heterogeneity is widely observed both in vivo and in vitro (Levaot et al. 2015; Søe et al. 2015). Additionally, the complexity of OCs makes them challenging to identify during osteoclastogenesis, as OCs are not the only multinucleated cells and lack specific markers in vivo(Miron et al. 2016), while pure OCs cannot be reliably obtained under normal in vitro conditions (Husch et al. 2021).

OCs play a crucial role throughout various stages of the bone formation process, including cavity development (Tosun et al. 2022), angiogenesis (Tosun et al. 2022; Xie et al. 2014), and remodeling (Durdan et al. 2022). The role of OCs in bone regeneration, such as fracture healing (Flick et al. 2003; Takeyama et al. 2014) and their potential in osteoinductive effects (Gamblin et al. 2014; Guo et al. 2021), has also attracted significant attention in recent years. Moreover, as one of the most important bone cell types, OCs play a crucial role in bone diseases. As such, therapeutic strategies targeting OCs are currently under intensive investigation.

In the field of bone biology, OCs remain a subject of ongoing research, with many questions still unanswered. Understanding the complexities of OC biology is not only essential for comprehending bone formation and development but also has significant implications for bone regeneration. This comprehensive review will gather current evidence on the origin of OCs, the OC fusion process, OC marker identification, and the pivotal roles OCs play in bone formation and regeneration, providing insights into their multifaceted contributions to skeletal tissue dynamics. Finally, therapeutic strategies for utilizing OCs in bone formation and regeneration in bone diseases are discussed.

## The origin of osteoclasts

### Origin of embryonic osteoclasts

As early as the 1970s, circulating mononuclear hematopoietic cells were identified as the precursors of OCs (Feng and Teitelbaum 2013; McDonald et al. 2021b). Later on, the well-established phenomenon of hematopoietic stem cell (HSC)-derived precursors fusing into multinucleated OCs, induced by macrophage colony-stimulating factor (M-CSF) and receptor activator of NF-κB ligand (RANKL), has further confirmed the origin of OCs (Husch et al. 2021). However, OCPs from HSCs do not form the earliest OCs in embryos. Recent studies (Jacome-Galarza et al. 2019; Yahara et al. 2020) broadened the knowledge of the origin and timing of OC occurrence. These studies indicate that erythromyeloid progenitors (EMPs) could also serve as a potential origin for tartrate-resistant acidic phosphatase positive (TRAP +) multinucleated OCs. During embryonic days 15.5–16.5 (E15.5–16.5), TRAP + multinucleated OCs were identified in Myb − / − mutant mice (Jacome-Galarza et al. 2019). The functional Myb gene is required for murine fetal hematopoiesis (Mucenski et al. 1991). Therefore, these TRAP + multinucleated OCs identified in Myb − / − mutant mice are derived from a source other than hematopoietic stem cells (HSCs). Primitive yolk-sac macrophages can undergo direct differentiation from EMPs in a Myb-independent transcriptional activator manner (Gomez Perdiguero et al. 2015). This observation implies that the earliest occurrence of embryonic OCs originates from EMP-derived precursors as early as E15.5–16.5. Furthermore, the observation of TRAP + multinucleated OCs at E16.5 in mouse embryos, in which osteoclastic progenitors derived from HSCs had been successfully eliminated, further confirms the previous results (Jacome-Galarza et al. 2019).

**Fig. 1:**
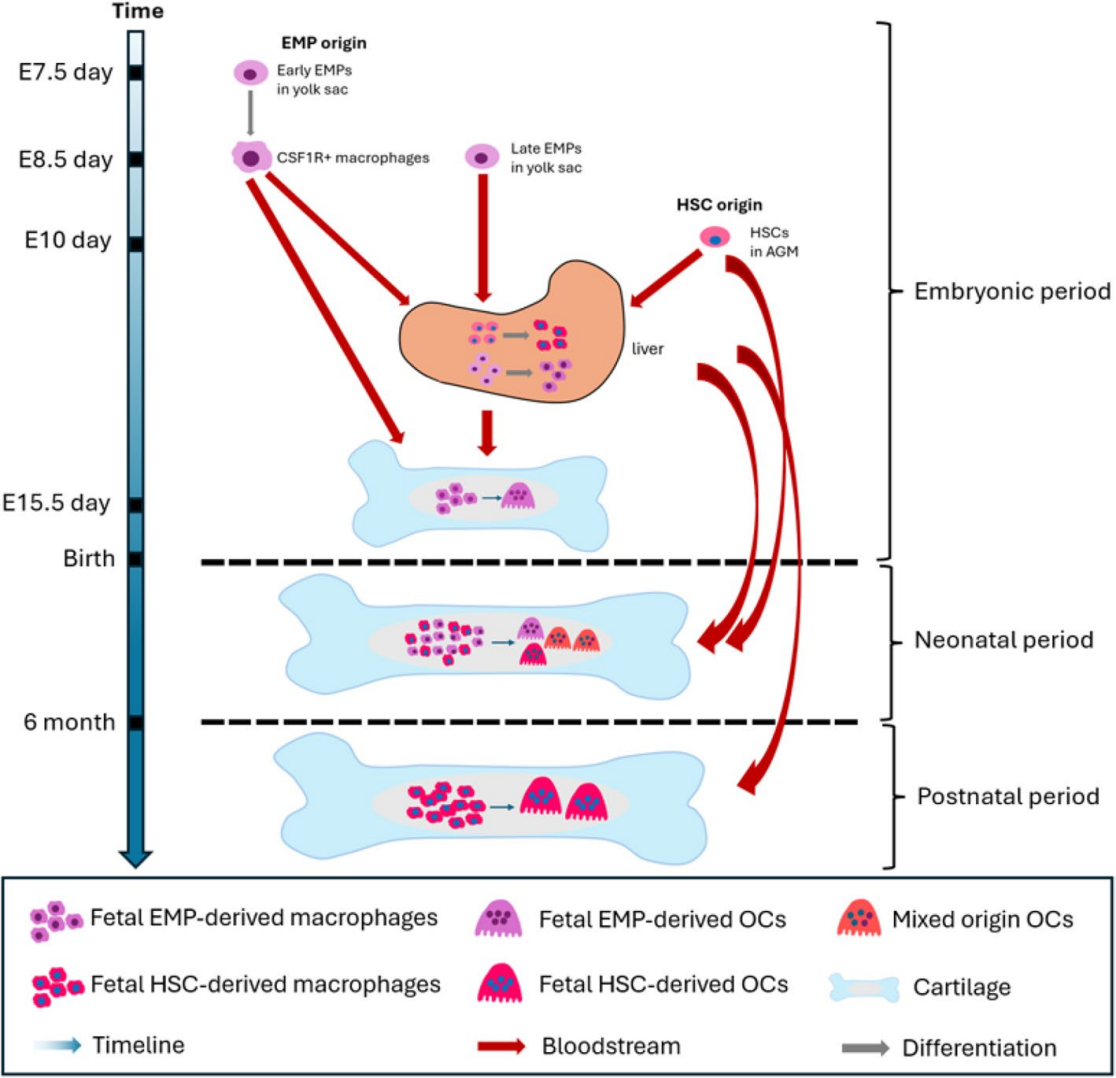
Schematic representation of the origin of osteoclasts in different pre- and postnatal life periods. Early erythromyeloid progenitors (EMPs) emerge at E7-7.5, giving rise to CSF1R + yolk sac macrophages. Late EMPs emerge at E8.25–9 and then migrate to the fetal liver and differentiate into osteoclast precursors (OCPs). These OCPs migrate to primary ossification centers, creating space for the bone marrow cavity. HSCs emerge at E10 eventually give rise to osteoclasts (OCs), participating in fetal bone marrow cavity formation together with EMP-derived OCs during neonatal period. During this period, EMP-derived OCs acquire one nucleus at a time from HSC-derived cells, creating mixed-origin OCs. Eventually, OCs from EMP and mixed origin are replaced by HSC-derived OCs

The precursor cells for monocytes/macrophages are predominantly generated through three successive waves of hematopoiesis. A comprehensive review of these three waves of hematopoiesis was provided recently by Yasuhito et al.(Yahara et al. 2022). The early and late EMPs emerge during the initial two yolk-sac waves of hematopoietic process (Boisset and Robin 2012). In short, the first wave of hematopoiesis starts around E7 within the blood island of the yolk sac. Early EMPs, produced by hemogenic endothelium, appear approximately between E7-7.5 and subsequently undergo direct differentiation into colony-stimulating factor 1 receptor (CSF1R) + primitive yolk-sac macrophages around E8.5, operating in a Myb-independent transcriptional activator manner. The late EMPs, Myb-dependent in their generation, arise from E8.25-E9 in the yolk sac and migrate to the fetal liver, where they transform into fetal liver monocytes. The final wave of hematopoiesis, occurring around E10.5, involves HSC precursor cells in the aorta-gonad-mesonephros (AGM) region, rather than arising from EMPs. Subsequently, HSCs migrate and colonize to the nascent fetal liver, mature and expand there, and finally colonize the bone marrow. These HSC-derived precursors also give rise to embryonic OCs and actively take part in the formation of bone marrow cavity with EMP-derived OCs around E17.5 (Jacome-Galarza et al. 2019) (Fig. 1).

### Origin of postnatal osteoclasts

Postnatally, HSCs gradually replace EMPs and play a critical role in the hematopoietic system throughout the rest of life (Jacome-Galarza et al. 2019; Yahara et al. 2020). The precursors of HSCs were observed in the yolk-sac and intra-embryonic AGM region at E10.5 (Medvinsky et al. 1993; Müller et al. 1994) (Fig. 1). Subsequently, these multilineage potent HSCs can differentiate into more lineage restricted progenitors and precursors, and further give rise to erythroid, myeloid, and lymphoid lineage mature cells, through a series of differentiational processes (Seita and Weissman 2010; Sun et al. 2021).

In the classical model of hematopoietic differentiation hierarchy, HSCs initiate the cascade by giving rise to multipotent progenitors (MPPs), which possess variable differentiation potential but lack self-renewal ability (Christensen and Weissman 2001). Progressing along the hierarchy, these MPPs undergo further differentiation into oligopotent progenitors, including common lymphoid progenitors (CLPs) (Serwold et al. 2009) and common myeloid progenitors (CMPs) (Akashi et al. 2000). Within the myeloid lineage, CMPs branch into megakaryocyte–erythrocyte progenitors (MEPs) (Nakorn et al. 2003) and granulocyte-monocyte progenitors (GMPs) (Pronk et al. 2007). Notably, GMPs, classified as oligopotent progenitors, subsequently undergo differentiation into mature cell types, such as granulocytes and monocytes (Pronk et al. 2007; Seita and Weissman 2010). These OCPs will migrate to bone resorption sites via the bloodstream, and there undergo fusion into OCs upon stimulation with M-CSF and RANKL produced by mesenchymal cells like osteoblasts (OBs) and osteocytes (Tsukasaki and Takayanagi 2019).

OC formation in adults can also arise from various other sources. Several studies have proposed that dendritic cells (DCs) can give rise to OCs in vitro in the presence of M-CSF and RANKL (Olsson et al. 2006), as well as under pathological conditions (Rivollier et al. 2004; Wakkach et al. 2008). However, there is no observed reduction in OC formation in the absence of DCs in mice. This suggests that DCs may not play a contributory role in the process of OC formation under normal physiological conditions (Kurotaki et al. 2019, 2014). A recent investigation into the stepwise cell fate decision-making during osteoclastogenesis, employing single-cell RNA sequencing (scRNA-seq), revealed the transient existence of CD11*c*-positive DC-like cells differentiated from the same murine bone marrow cells as OCs. Moreover, the same researchers used CD11*c*-Cre to delete the *RANK* gene, leading to *RANK* depletion in DCs and observed a substantial reduction in OC formation both in vitro and in vivo (Tsukasaki et al. 2020). These results suggest that the monocyte-origin hypothesis and the DC-origin hypothesis are not mutually exclusive, as DC-like cells share the same origin as OCs, and that DC can be a transitional state during osteoclastogenesis. This also explains why, under normal physiological conditions, the absence of DCs does not affect the formation of OCs.

Moreover, under continuous soluble-RANKL stimulation, OCs undergo fission, dividing into motile smaller daughter cells known as osteomorphs (McDonald et al. 2021a). scRNA-seq analysis revealed that osteomorphs exhibit a distinct genetic profile compared to OCs and macrophages. These daughter cells have the capability to undergo fusion either with multinucleated OCs or among themselves, thereby recreating new functional OCs. This suggests that osteomorphs could serve as a source of OCs.

Furthermore, tissue-specific macrophages (Gomez Perdiguero et al. 2015) can contribute to OC formation. Interestingly, these tissue-specific macrophages are initially derived from yolk sac EMPs, migrate to different tissues where they differentiate into macrophages during embryonic development, and are later replaced by HSC-derived cells (Gomez Perdiguero et al. 2015). It has also been reported that other cells, such as pro- and pre-B lymphocytes (Khass et al. 2019; Manabe et al. 2001), and embryonic stem cells (Nishikawa et al. 2014), can differentiate into OCs. However, these latter cell types are not considered as a major source for OC generation under normal physiological or pathological conditions.

### Mixed origin osteoclasts

In the neonatal period, OCs nuclei can originate from both EMPs and HSCs. Following the third wave of hematopoiesis, HSCs gradually replace EMPs as the primary source of OCs (Yahara et al. 2022). By generating *Csf1r*^*cre*^;*Rosa26*^*LSL−YFP*^ and *Csf1r*^*cre*^;*Rosa26*^*LSL−tdTomato*^ mice, YFP and tdTomato fluorescence can specifically label cells expressing *Csf1r*, including OCPs such as macrophages. Conducting a time-course parabiosis experiment and surgically connecting these mice for 4–8 weeks of blood sharing (Jacome-Galarza et al. 2019), all the OCs in both parabionts co-express YFP and tdTomato. This suggests that EMP-derived OCs can acquire OCPs from both partners through blood circulation. During this period, OCPs in circulation originate from HSCs, implying that OCs in this specific timeframe are derived from both EMPs and HSCs (Fig. 1).

### Cell fusion based on heterogeneity

**Fig. 2:**
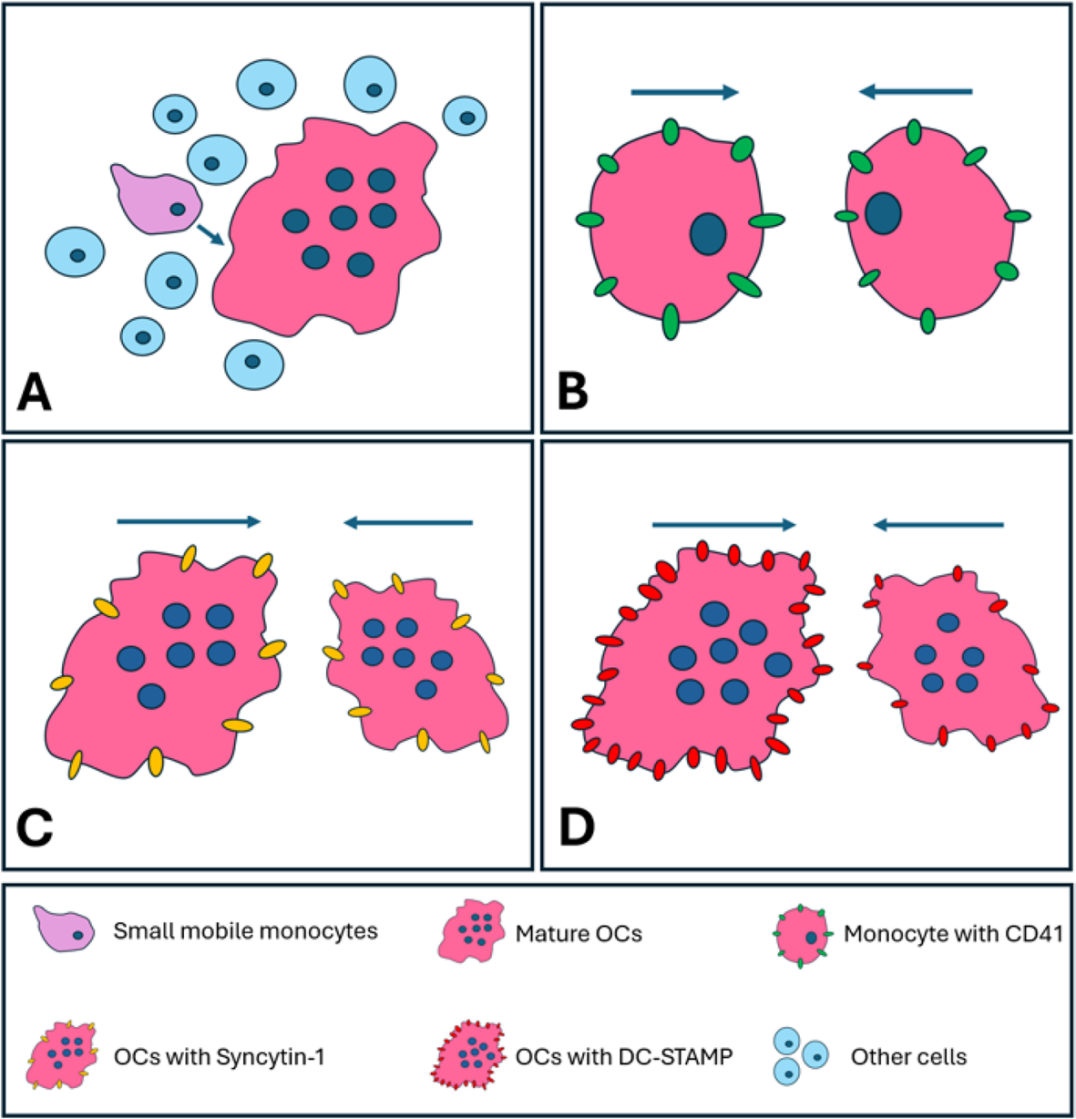
Schematic representation of the fusion modes that form OCs. **A**. Mononucleated cell fuse with multinucleated cells. Cells with fewer nuclei are less mature and exhibit greater mobility compared to larger multinucleated cells. **B**. Mononucleated cell fuse with mononucleated cells. Transmembrane protein CD47 is predominantly expressed by small OCPs or OCs with few nuclei. **C**. Multinucleated cells fuse with multinucleated cells. In the later stages of OC differentiation, fusion between multinucleated cells are regulated by transmembrane protein Syncytin-1. **D**. Fusion of OCPs demonstrates a heterogeneous profile for DC-STAMP. Fusion occurrs between DC-STAMP^lo^ cells and DC-STAMP^hi^ cells

In the process of OC formation, OCPs form into multinucleated and giant OCs by cell fusion. This intricate fusion process involves sequential events: (1) cell attraction/migration, (2) recognition of fusion partners, (3) cell–cell adhesion, and (4) fusion of plasma membranes (Fig. 2). The success of OC fusion depends on the heterogeneity of the fusion partners, including differences in nucleus number, mobility, and the expression of particular surface proteins.

A small subset of OCPs known as "fusion founders," have been identified as capable of fusing with “followers,” with only 2.4% of OCPs acting as initiators of cell fusion (Levaot et al. 2015). It was also observed that nearly 70% of multinucleated OCs fused with mononucleated OCPs in OC culture, indicating a preference for more mature OCs to fuse with a less mature pre-OC. Additionally, 62% of fusion events occurred between mobile and immobile partners (Søe et al. 2015). Typically, smaller cells exhibit greater mobility compared to larger multinucleated cells (Fig. 2A). In neonatal period, OCs also fuse with the mononucleated OCPs to sustain their maintenance (Jacome-Galarza et al. 2019). Quiescent OCPs, lacking proliferation potential, play an essential role in OC precursors fusion and OC maturation. Studies conducted both in vivo and in vitro indicate that the presence of quiescent OCPs may enhance OC formation (Lee et al. 2015; Takahashi et al. 2010).

The fusion of OCPs is a complex process that entails the engagement of various cell surface receptors. Dendritic cell-specific transmembrane protein (DC-STAMP) is a 53 kDa cell surface protein that has 7 transmembrane regions (Chiu et al. 2012). Its primary expression is observed in cells of the monocyte/macrophage lineage, including myeloid dendritic cells (Hartgers et al. 2000). Recognized as a master regulator in the osteoclastogenesis process, DC-STAMP plays a pivotal role in the fusion of mononucleated OCPs, leading to the formation of multinucleated OCs (Chiu and Ritchlin 2016). It was found that fusion partners of OCPs demonstrate a heterogeneous profile for DC-STAMP (Mensah et al. 2010). Mononuclear OCPs expressing low levels of exhibited the ‘‘master fusogenic’’ phenotype, and cell fusion exclusively occurred between DC-STAMP^lo^ cells and DC-STAMP^hi^ cells (Mensah et al. 2010) (Fig. 2D).

Furthermore, CD47, also referred to as integrin-associated protein, has been identified in association with integrin αvβ3 (Brown and Frazier 2001). The heterogeneity in CD47 expression contributes to OC fusion. CD47 is predominantly expressed by small OCPs or OCs containing few nuclei (Hobolt-Pedersen et al. 2014; Maile et al. 2011) (Fig. 2B). As OCs mature and nuclei increase, the expression of CD47 decreases, suggesting a role for CD47 in promoting the early cell fusion of mononucleated OCPs (Møller et al. 2017).

Another relevant cell–cell fusion protein is syncytin, which is a captive retroviral envelope protein, possibly involved in the formation of the placental syncytiotrophoblast layer generated by trophoblast cell fusion at the maternal–fetal interface (Gong et al. 2005). Syncytin-1 and its receptor amino acid transporter 2 (ASCT2) are expressed by OCs (Soe et al. 2011) and involved in OCPs fusion (Møller et al. 2017). Interestingly, CD47 and Syncytin-1 play distinct roles in different stages of OC differentiation. CD47 primarily influences the fusion of mononucleated cells or cells with few nuclei during the early stages of OC differentiation. In contrast, Syncytin-1 predominantly affects the fusion of multinucleated cells (more than 2 nuclei) during the later stages of OC differentiation while inhibiting the fusion of mononucleated OCP cells (Møller et al. 2017) (Fig. 2C).

Moreover, the molecular mechanism of OCs fusion involves interactions among various cellular and molecular factors. In addition to the factors mentioned above, OC-STAMP (Khan et al. 2013), ATP6v0d2 (Lee et al. 2006), protocadherin-7 (Nakamura et al. 2014), E-cadherin (Fiorino and Harrison 2016), CD9 (Ishii et al. 2006), and CD109 (Wang et al. 2013) are involved in OC fusion during OC differentiation. However, the association between OC fusion and heterogeneity of these factors remains unclear.

## Cellular characteristics change during the transition from precursor to osteoclast

The transition from precursor cells to OCs is a dynamic process characterized by cellular phenotypic/morphological changes. This section provides an overview of the cellular characteristics that undergo alterations during the transition from monocytes/macrophages to OCs in a chronological sequence, focusing mainly on morphology and key markers associated with osteoclastogenesis. To clarify the concept, only markers expressed within the macrophage lineage are considered here (Table 1). However, no specific protein markers are exclusively expressed on pre-OCs in the monocytes-OC axis. All markers expressed on pre-OCs are also expressed on OCs, often with stronger signals. After OC maturation, specific characteristics gradually emerge. It should be noted that no single marker is capable of identifying OCs, and there is also not any single marker specifically expressed by OCs. Consequently, we recommend that when identifying OCs in vivo, it is necessary to use at least two OC markers or consider the cell environment (e.g. bone surface) for accurate determination.

**Table 1:** The change of cellular characteristics during the transition from precursor to OC

Cell Surface Marker	Monocytes/Macrophages	Pre-OCs	Mature OCs
			Multinuclearity
CD14	+ +		
CD47	+ +		
CD68	+ +		
F4/80	+ +		
CD44		+	+ +
RANK		+	+ +
OSCAR		+	+ +
TRAP		+	+ +
CTR		+	+ +
CAII		+	+ +
CTSK			+ +
MMP-9			+ +
Integrin β3/CD61			+ +
ATP6V0D1			+ +

For table purposes, + : expressed in cell-type: +  + : highly expressed in cell-type

### Monocytes/macrophages

#### CD14

CD14 is primarily expressed and produced by monocytes/macrophages, making it a reliable marker for these cells (Ziegler-Heitbrock and Ulevitch 1993). However, CD14 undergoes down-regulation during the differentiation of macrophages into pre-OCs (Takeshita et al. 2000). Consequently, CD14 is widely used in the field of depicting the conversion process from monocytes/macrophages to OCs (Husch et al. 2021).

#### CD47

CD47, a transmembrane protein, plays a pivotal role in regulating diverse cellular functions such as apoptosis, proliferation, adhesion, and migration (Cham et al. 2020; Hayat et al. 2020; Sick et al. 2012; Soto-Pantoja et al. 2015). It is reported that CD47 primarily express in small OCs and mononucleated pre-OCs, and decreased in the process of fusion (Hobolt-Pedersen et al. 2014; Møller et al. 2017), and play an important role in promoting OC formation both in vivo and in vitro (Lundberg et al. 2007; Møller et al. 2017). It is worth noting that CD47 is expressed on the pre-OCs situated on collagen, rather than on mineral surfaces (Søe et al. 2019).

#### F4/80

F4/80 is a well-established marker for macrophages in murine tissue (Dos Anjos Cassado, 2017). Upon differentiation of myeloid-lineage cells into macrophages, F4/80 is synchronously expressed (Deng et al. 2022). However, it is worth noting that F4/80 is rapidly down-regulated in the early stages of osteoclastogenesis and is not typically expressed as a marker for OCs (Lean et al. 2000).

### Pre-OCs

#### CD44

CD44 serves as a cell surface receptor expressed on numerous cells, playing a pivotal role in regulating cell adhesion and migration (Senbanjo and Chellaiah 2017; Sterling et al. 1998). CD44 is widely reported to be expressed on the surfaces of OCPs and OCs (Kania et al. 1997; Samanna et al. 2007). Its expression on OCPs is upregulated during their transition to OCs (Li et al. 2015). In vitro, utilizing a CD44 antibody on OCPs inhibits the formation of OCs in a dose- and time-dependent manner (Kania et al. 1997). In vivo, deficiency in CD44 results in the impaired function of OCs (Li et al. 2015). Additionally, there are reports of CD44 expression on multinucleated giant cells (MNGCs) (Bonnema et al. 2003; McFarlane and Revell 2004).

#### Receptor activator of nuclear factor kappa-B (RANK)

RANK is a transmembrane signaling receptor expressed on the surface of hematopoietic cells. It serves as a key regulator in osteoclastogenesis and OC activities through the RANK/RANKL pathway (Boyle et al. 2003). Subtypes of CD14 + peripheral blood mononuclear cells (PBMNCs) with high levels of RANK expression give rise to OCs in roughly double the numbers compared to their counterparts with middle or low expression levels (Atkins et al. 2006). Even after OC formation, RANK continues to regulate OC maturation and activation by inducing actin ring formation, ultimately resulting in increased osteoclastic bone resorption (Boyce and Xing 2008). However, RANK is not expressed on MNGCs (McNally and Anderson 2011). Consequently, RANK could be used to distinguish pre-OCs and OCs from MNGCs.

#### Osteoclast-associated receptor (OSCAR)

OSCAR belongs to the family of leukocyte receptor complex proteins, primarily associated with OCs and plays a significant role in OC differentiation and function (Kim et al. 2002). It was firstly discovered on the surface of murine pre-OCs and mature OCs, with no expression detected on macrophages or dendritic cells (Kim et al. 2002). However, OSCAR is not only expressed on human OCs, but also in other cell types like monocytes/macrophages and dendritic cells (Merck et al. 2004). Therefore, OSCAR could be used as a marker to identify and characterize OCs in specific biological contexts.

#### Tartrate-resistant acid phosphatase (TRAP)

TRAP is a well-known histochemical marker expressed by OCs, which is mainly localized within the ruffled border area and secreted during bone resorption (Ljusberg et al. 2005). Consequently, TRAP represents an important marker for bone-resorbing OCs maturity and functionality. However, some reports indicate that TRAP expression appears to be largely independent of resorption  (Rucci et al. 2019; Susa et al. 2004). TRAP expression can also be detected in immature dendritic cells (Hayman et al. 2001) and mononuclear pre-OCs (Xie et al. 2014), meaning that TRAP is not exclusively expressed by mature OCs. Interestingly, MNGCs with the inability of biomaterial resorption also express TRAP at a low level (Barbeck et al. 2016). This observation adds a layer of complexity to the interpretation of TRAP expression by cells, especially in the context of identifying OCs in vivo. Other conditions for identifying OCs, such as bone environments, need to be integrated. However, TRAP is a reliable marker for OCs cultured in vitro. The expression of the TRAP gene is notably strong in mature OCs and relatively weaker in mononucleated pre-OCs, whereas bone marrow macrophages do not exhibit expression of these genes (Takeshita et al. 2000). It is widely acknowledged that TRAP staining tends to be positive primarily after the conversion of monocytes/macrophages into mononucleated pre-OCs and multinucleated mature OCs (Boyle et al. 2003; Takeshita et al. 2000; Zhu et al., 2018).

#### Calcitonin receptor (CTR)

CTR, a G-protein-coupled receptor, regulates OC activity through its binding to calcitonin (Dacquin et al. 2004). Acting as a specific marker, CTR aids in distinguishing OCs from the diverse cell populations generated during osteoclastogenesis. Its exclusive expression on pre-OCs and OCs makes CTR one of the most reliable markers for distinguishing OCs from macrophages and their polykaryons in mammals (Lee et al. 1995; Quinn et al. 1999). However, CTR is not expressed in avian OCs (Nicholson et al. 1987). Additionally, CTR may also be expressed on the other cell in bone environment such as chondrocytes (Sondergaard et al. 2010) and osteocytes (Gooi et al. 2010).

#### Carbonic anhydrase II (CAII)

CAII is an enzyme belonging to the carbonic anhydrase family, and it plays a crucial role in OC activity by participating in the acidification of the resorption lacunae (David et al. 2001). Immunohistochemical staining has demonstrated strong CAII expression in OCs, while foreign body giant cells, peritoneal macrophages, lung macrophages, and cultured peripheral monocytes have shown negative staining (Sundquist et al. 1987). Another study supports this by showing that the CAII gene is strongly expressed in mature OCs and weakly expressed in pre-OCs, while it is not expressed in monocytes/macrophages (Takeshita et al. 2000). Specifically, gene expression of CAII is up-regulated in OCs when they begin to resorb bone (Asotra et al. 1994), which is consistent with the observation that CAII is only expressed in OCs and is involved in their acidification activity (David et al. 2001; Sundquist et al. 1987). Consequently, CAII can serve as a reliable OC marker in the process of osteoclastogenesis.

### OCs

#### Multinuclearity

Multinuclearity emerges as a prominent phenomenon and can be easily observed during the conversion of OCPs into OCs (Husch et al. 2021). In vitro induction of OC formation using osteoclastic formation cytokines, i.e. M-CSF and RANKL, can lead to the development of OCs with dozens of nuclei, possibly influenced by differences in substrate composition compared to living bone (Jain and Weinstein 2009). It is noteworthy that multinuclearity is not exclusive to OCs; foreign body giant cells and Langerhans giant cells also exhibit this characteristic (Ahmadzadeh et al. 2022).

#### Cathepsin K (CTSK)

CTSK is secreted by OCs to facilitate type I collagen degradation during the bone resorption process (Wilson et al. 2009). It is a highly expressed marker in the late stages of osteoclastogenesis, corresponding to the formation and resorption functioning of mature OCs (Drake et al. 1996). However, it is worth noting that the expression of CTSK, even in conjunction with TRAP expression on the same cell, does not always indicate the presence of OCs. It can also be a expressed in MNGCs (Park et al. 2013) and in osteocytes during lactation (Qing et al. 2012). Nevertheless, the presence of CTSK is minimal within MNGCs (Khan et al. 2014).

#### Matrix metalloproteinase 9 (MMP-9)

MMP-9, also known as matricin, is a protein secreted by highly activated OCs that plays a role in the breakdown of the extracellular matrix (Grassi et al. 2004), as well as OC migration (Samanna et al. 2007). MMP9 has been observed to have weak expression on monocytes/macrophages, with its expression increasing as OCPs develop into mature bone resorbing OCs (Kusano et al. 1998). However, MMP-9 cannot serve as a specific marker for OCs, as it is also expressed by numerous other cell types, including neutrophils, macrophages, fibroblasts, and breast cancer cells (Yabluchanskiy et al. 2013; Yousef et al. 2014). Therefore, MMP9 serves more like an indicator for assessing the functionality of OC resorption.

#### Integrin β3

Integrins are transmembrane proteins that play a crucial role in cell–cell and cell-extracellular matrix (ECM) adhesion (Hynes 2002). Mature OCs express four different integrin dimers: αvβ3 (Deng et al. 2021), α2β1 (Helfrich et al. 1996; Rucci and Teti 2016), αvβ1 (Helfrich et al. 1996), and α9β1 (Rao et al. 2006). Upon exposure of OCPs to RANKL and the initiation of the biological cascade of osteoclastogenesis, integrins αvβ5, as well as αvβ2 (also known as CD51/18), expressed on bone marrow macrophages or their polykaryons, disappear and are replaced by αvβ3 (also known as CD51/61), which is highly expressed on OCs (Deng et al. 2021; McHugh et al. 2000; Zhang et al. 2022). Therefore, integrin β3 could be considered as a reliable marker for identifying OCs.

#### ATP6V0D1

Vacuolar ATPase (V-ATPase) is a giant molecule present in the plasma membrane of a wide range of cells, including kidney intercalated cells, OCs, macrophages, neutrophils, sperm, and certain tumor cells (Izumi et al. 2003). V-ATPase has two main parts: the extracellular V1 domain and the membrane-bound V0 domain. Moreover, Subunit d in the V0 domain has two isoforms, D1 and D2 (Qin et al. 2012). ATP6V0D1, also known as vacuolar-type proton pump-3 (Vpp3), can be used as a reliable OC marker in vivo within the bone environment and is undetectable in circulating cells in the bone marrow cavity (Romeo et al. 2019).

## Osteoclast function in bone formation

### The role of osteoclasts in bone marrow cavity formation

Bone formation in embryonic skeletal development occurs via either intramembranous or endochondral ossification. Endochondral ossification, characterized by an intermediate cartilage stage, serves as the predominant process in embryonic skeletal development (Salhotra et al. 2020). The bone marrow plays a central role in the processes of hematopoiesis and immune system regulation (Muguruma et al. 2006). After the invasion of vessels into the cartilage, OCPs enter the central region of hypertrophic cartilage through the bloodstream, subsequently fusing into OCs and contributing to the formation of the bone marrow cavity by removing hypertrophic chondrocytes and resorbing the calcified cartilage matrix (Salhotra et al. 2020; Sivaraj and Adams 2016). In *Rank-deficient* mice, OCs were shown to be eliminated, while monocytes/macrophages within the bone marrow cavity were significantly increased. In *Pu.1-deficient* mice, both OCs and monocytes/macrophages were deleted. Both types of deficient mice exhibited a delayed formation of bone marrow cavities, accompanied by an extension of the hypertrophic chondrocyte zone (Tosun et al. 2022). This suggests that OCs, rather than macrophages, play a crucial role in cartilage resorption and the creation of these cavities. Although, the formation of bone marrow cavities was delayed, they still formed despite the absence of OCs. This suggests that OCs are not a prerequisite but play a partial role in the formation of bone marrow cavities (Tosun et al. 2022). However, The studies conducted by Jacome-Galarza et al. (Jacome-Galarza et al. 2019) showed that the OCs seems indispensable in bone marrow cavity formation. They generated *Tnfrsf11a*^*cre*^*;Csf1r*^*fl/fl*^ mice, which lack EMP-derived macrophages while leaving HSCs and blood cells unaffected. Consequently, these mice were characterized by a lack of EMP-derived embryonic OCs, while HSC-derived OCs will emerged in their later life. They found that these mice exhibited a severe osteopetrotic phenotype in early stage, including initially lack of bone marrow formation (Jacome-Galarza et al. 2019). Nonetheless, OCs are not always the cells for cartilage resorption. Endothelial cells (ECs) in H-type vessels have been reported to secret MMP-9, which resorb growth plate cartilage, leading to directional bone growth (Romeo et al. 2019). However, the authors overlooked the differentiation between OCs and chondroclasts, but uniformly considered these cells as OCs. Beyond the substrate disparity, there are few distinctions between OCs and chondroclasts in terms of cellular structure and behavior, leading many to consider these two cell types as essentially the same (Odgren et al. 2016). However, using comparative transcriptomics analysis, differential molecular profiles of the two cell types were established (Khan et al. 2020). Moreover, postnatally, osteopetrosis manifests with an OC-poor phenotype that displays reduced marrow cavity formation (Wu et al. 2017). This also suggests a role of OCs in bone marrow cavity maintenance.

### The role of osteoclasts in angiogenesis

From the earliest stages of embryonic bone development, the process of osteogenesis remains complicatedly coupled with angiogenesis, extending throughout the entirety of lifelong bone remodeling (Sivaraj and Adams 2016). Interestingly, emerging evidence has shown that OCs have an intimate relationship with ECs and angiogenesis. Results from an in vitro study have demonstrated that conditioned medium from human OC cultures stimulates blood vessels formation (Tanaka et al. 2007). However, macrophages are also proven to possess pro-angiogenic characteristics (White et al. 2004). The findings of this study remain inconclusive because the heterogeneous OC culture still contains a significant number of macrophages. It is reported that using osteoprotegerin (OPG) to suppress osteoclastogenesis in vivo results in a dose-dependent inhibition of angiogenesis, implying that OCs play a role in promoting angiogenesis (Cackowski et al. 2010). Conversely, induction of osteoclastogenesis through RANKL led to an increase in calvarial vessel density (Cackowski et al. 2010). Several studies have indicated that angiogenesis stimulation during osteogenesis and fracture repair is mainly caused by OC-secreted matrix MMP-9 (Cackowski et al. 2010; Colnot et al. 2003; Isowa et al. 2010). Furthermore, OCs safeguard neighboring ECs from senescence by secreting angiogenin (ANG), thereby preserving their proliferative activity (Liu et al. 2021).

The intimate relationship between angiogenesis and osteogenesis is highlighted by the existence of a specific vessel type known as H-type vessels, which play a crucial role in coupling these processes (Kusumbe et al. 2014). Remarkably, H-type vessels are predominantly located in the rapidly growing bone region, named metaphysis, and play a pivotal role in coupling of angiogenesis to osteogenesis (Peng et al. 2020; Xie et al. 2014). A specific OC subsets, called vessel-associated OCs, reside in the bulge and arch structures of H -type capillaries (Romeo et al. 2019). These OC subsets are reported playing a role of regulating anastomoses of H-type vessels (Romeo et al. 2019). The ECs on H-type vessels, instead of OCs, are responsible for secreting MMP-9 and resorbing cartilage to lead directional bone growth. Importantly, disrupting the orientation of H-type vessels by misdirecting them results in contorted bone shape (Romeo et al. 2019). Moreover, The expression levels of significant osteoclastogenic factors, such as CSF1, Il-1α, and TNFRSF11a, were markedly elevated in H-type vessel ECs, and endothelial specific loss of Tnfsf11a reduced the OC numbers (Romeo et al. 2019). These suggest that OCs and H-type vessels are indispensable for each other. Furthermore, pre-OCs, defined as TRAP + mononuclear cells, were reported to have the capacity of producing platelet-derived growth factor-BB (PDGF-BB) to induce the formation of H-type vessels (Xie et al. 2014). The pro-angiogenic factors triggered by OCs, such as vascular endothelial growth factors (VEGFs) released from the bone matrix by OCs, are recognized for their pivotal roles in both ECs (Bergers et al. 2000; Olsson et al. 2006) and OC function (Engsig et al. 2000; Olsson et al. 2006). The inhibition of VEGF has been observed to impede OC invasion into hypertrophic cartilage, indicating the significance of VEGF in OC invasion activities and normal bone development (Engsig et al. 2000).

Endochondral angiogenesis is known to start with blood vessel invasion primarily stimulated by the hypertrophic chondrocytes. OCs are conventionally considered to initiate their essential functions only after their precursors had migrated to the primary ossification center through circulation (Salhotra et al. 2020; Sivaraj and Adams 2016). Notably, observing from the results of Emcn immunostaining, OC-deficient mice exhibited a postponed vascular invasion during endochondral ossification (Tosun et al. 2022). This suggests a collaborative effort of hypertrophic chondrocytes and OCs on the initial blood invasion stage. However, OCs alone lack the capability to induce angiogenesis in endochondral ossification. A *Csf-1* mutation in mice causes severe OC-poor osteopetrosis, showing absence of both tooth eruption and invading vessels (Dobbins et al. 2002; Jacome-Galarza et al. 2019). Systemic intraperitoneal injections of CSF-1 from birth in *Csf-1* mutation mice restored the functional OC population, teeth eruption and decreased the bone density, but failed to restore vessel invasion (Iizuka et al. 1992; Marks et al. 1997). It seems likely that OCs can promote angiogenesis rather than initiate the vascularization process during embryonic bone development.

### The role of osteoclasts in bone remodeling

Bone remodeling is a continuous and dynamic process throughout life, which orchestrates OCs to resorb and OBs to form bone in a spatiotemporal manner to replace old bone, maintain bone homeostasis, repair micro-bone damage, and adjust bone strength to physical requirements (Durdan et al. 2022). Once the balance between OCs and OBs is broken, either osteoporosis or osteopetrosis will occur, resulting in low bone quality. Interestingly, bone resorption is decreased in OC-rich osteopetrosis, yet formation is increased (Thudium et al. 2014), while bone resorption and formation activities are both decreased in OC-poor osteopetrosis. In these cases, OCs seem to have a pro-osteogenic effect on OBs and their precursor cells.

**Fig. 3:**
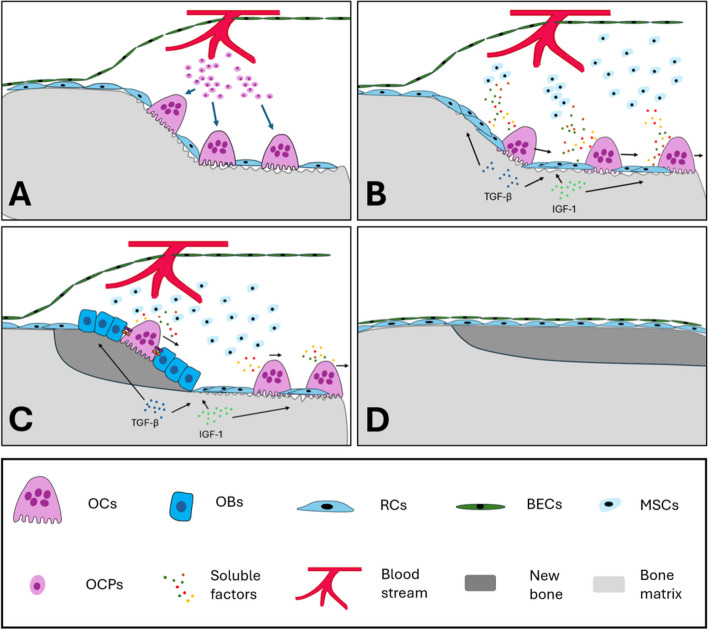
Schematic representation of the bone remodeling process in the basic multicellular unit (BMU). **A**. OCPs from the bloodstream circulation come to the bone surface and initiate resorption activity, causing bone marrow envelop cells (BECs) to lift up and form a BMU. **B**. OCs move forward, initiating the bone-forming process by passively releasing bone matrix-derived factors and actively releasing soluble factors and/or EVs. Through these mechanisms, OCs stimulate OB lineage cell migration and induce angiogenesis, finally promoting bone formation. Among these processes, the most important step is OCs stimulating the expansion of reversal cells (RCs), leading to an increase in the density of RCs. Then, the reversal cells transition from the pro-resorption phase to the pro-osteogenic phase, initiating osteoblastogenesis. **C**. Several waves of resorption occur during bone remodeling, with OCs reappearing on the bone surface, mixing with RCs and OBs, and interacting with them via membrane-binding proteins. **D**. Bone remodeling is completed

Reversal cells (RCs), a population of osteoblast lineage cells, appear as elongated cells with flattened nuclei on the bone surface (Abdelgawad et al. 2016). At the early stages of bone remodeling, these cells surprisingly support the OC resorption activity by secreting MMP13 (Andersen et al. 2004) (Fig. 3A). Later, this group of cells switches into a pro-osteogenic phenotype in the reversal phase, which is the key step to transition bone resorption to formation in the bone remodeling process (Lassen et al. 2017). The initiation of this process is reported to have a high relevance to the density of RCs. When at least 75% of the eroded surface is covered by RCs, sequential osteogenesis will be initiated (Jensen et al. 2015, 2012). OCs play the key role in driving RC expansion to increase their cell density and switching pro-resorption RCs to a pro-osteogenic phenotype to initiate the bone-forming reversal phase (Fig. 3B). One potential mechanism behind this could be that when OCs resorb bone, immobilized factors such as transforming growth factor-β (TGF-β) (Oursler 1994) and insulin-like growth factor 1 (IGF1) (Xian et al. 2012) are released from the bone matrix. These factors are proven to induce mesenchymal stem cell (MSC) migration and osteogenic differentiation (Oursler 1994; Xian et al. 2012). 97% of the RCs have been shown to be positive for the OB marker RUNX2 (Andersen et al. 2013). Further study indicated that these RCs could differentiate into bone-forming OBs during the reversal phase (Ichida et al. 2011; Nakashima et al. 2002). Interestingly, several OC-mediated resorption waves were observed in the bone remodeling process (Lassen et al. 2017). This suggests that after colonization of the eroded surface by RCs, OCs reappear and mix with RCs, casting their impact on increasing the RC population and osteogenic stimulation, and stopping resorption until reaching the threshold for initiating osteogenesis process (Lassen et al. 2017).

Most schematic drawings depict bone remodeling as a series of distinct steps (i.e. resorption, reversal phase, bone formation Charles and Aliprantis 2014; Durdan et al. 2022; McDonald et al. 2021b; Sun et al. 2021). In reality, the steps in the processes of bone resorption and formation occur likely with no strict start and ending, but smoothly transitioning into one another. Moreover, these processes are characterized with several overlapping resorption and formation waves, allowing OCs and OBs to co-localize (Lassen et al. 2017). As a result, proteins on the membranes of involved cell types can interact, activating various signaling pathways (Fig. 3C). For instance, Ephrin B2 (EFNB2) on OCs can bind to EFNB4 on OBs. Activating this Ephrin signaling pathway can either suppress OC differentiation (via reverse signaling) or promote OB differentiation while preventing its apoptosis (via forward signaling) (Tonna et al. 2014). Moreover, FAS Ligand (FASL)-FAS (Wang et al. 2015) and Semaphorin 3A (SEMA3A)-NRP1 (Hayashi et al. 2012) between OCs and OBs are also critical bidirectional communication molecules acting on signaling pathways to regulate OC and OB activities.

The mostly investigated coupling factors are those secreted by OCs (Fig. 3B, C), such as, Semaphorin 4D (SEMA4D) (Negishi-Koga et al. 2011), Cardiotrophin-1 (CT-1) (Walker et al. 2008), Sphingosine 1 Phosphate (S1P) (Lotinun et al. 2013), Collagen Triple Helix Repeat Containing 1(CTHRC1) (Takeshita et al. 2013), and Complement Component Ca (C3a) (Matsuoka et al. 2014). Moreover, OCs can secrete extracellular vesicles (EVs) such as exosomes (Ikebuchi et al. 2018), microvesicles (Sun et al. 2021), and apoptotic bodies (Ma et al. 2021), which contain soluble factors or microRNAs cargo targeted towards nearby or more distant OBs. These interactions between OCs and OBs have been extensively reviewed (Charles and Aliprantis 2014; Durdan et al. 2022; McDonald et al. 2021b; Sun et al. 2021). Recently, our team revealed that mature OCs secrete EVs as a protein cargo to promote osteogenic differentiation of MSCs in vitro and further validated the bone-forming efficacy of OCs and their secreted EVs in mouse tibial bone defects. By employing proteomic and functional analysis, we demonstrated that thrombin-cleaved phosphoprotein 1 (SPP1) in OC-secreted EVs is particularly responsible for initiating the differentiation of MSCs into OBs by activating signaling pathways involving TGFβ1 and Smad family member 3 (SMAD3) (Faqeer et al. 2023). All the evidence mentioned above provides insight into the role of OCs in promoting bone formation.

In summary, OCs precede the appearance of OBs in the bone remodeling process (Fig. 3A, B). Moreover, OCs initiate the bone remodeling process and play a critical role in the subsequent bone-forming phase. This OC-mediated bone-forming process explains why bone formation occurs in a site-specific manner, achieving spatiotemporal coupling of resorption to bone formation. Given the fact that OCs also precede bone formation in material-induced bone regeneration (Guo et al. 2021), OCs could play a similar role as it in bone remodeling process.

## Osteoclasts in bone regeneration

### The role of osteoclasts in bone fracture repair

Bone fracture healing constitutes a multifaceted process requiring the orchestrated interplay of diverse cascades, often marked by the sequential occurrence of four overlapping phases: inflammation, revascularization after destruction of vessels, bone formation and continuous bone remodeling (Claes et al. 2012). Both increased OB and OC activities are required in this healing process as rapid bone formation, as well as bone remodeling and callus resorption is needed (Zhang et al. 2023a). In primary bone healing, OCs bridge the two sides of the fractures by forming tunnels called cutting cones that facilitate the in-growth of blood vessels. This, in turn, enables the recruitment of bone-forming precursors to the fracture sites, where they undergo further differentiation to bone forming OBs (Einhorn 2005). Secondary bone healing is the most common process of bone healing that bridges larger defect gaps, characterized by an intermediate stage of cartilage formation to produce a soft callus, followed by the development of woven bone to create a hard callus (Claes et al. 2012). The role of OCs in soft callus remodeling remains controversial, as some evidence shows that OCs may be redundant, while other evidence demonstrates they are not (Flick et al. 2003). Later, OCs and OBs orchestrate the process of resorption and bone formation at the hard callus and bone remodeling stages (Zhang et al. 2023a).

Genetic or pharmacological depletion of OCs has been used to investigate their role in bone healing (Table 2). RANKL KO mice showed a significant decrease in OC numbers, leading to diminished soft callus and hard callus resorption, which ultimately resulted in impaired bone healing (Flick et al. 2003). The authors suggested that delayed bone healing in these RANK KO mice might be due to fewer blood vessels. As discussed in the previous section on the effect of OCs on vascularization, the lack of OCs could have contributed to the reduced blood vessel formation. Moreover, treatment with clodronate liposomes in femur fracture mice to deplete OCPs and reduce OC numbers and activity led to delayed resolution of callus cartilage (Lin and O'Connor 2017). In contrast, administration of the cathepsin K inhibitor odanacatib (Pennypacker et al. 2016) or genetical depletion of CTSK (Gentile et al. 2014) in mice fracture model increased number of cathepsin K positive OCs in the callus, resulting in enhanced mineralized bony tissue and significantly reduced residual cartilage. However, The therapeutic application of RANK signaling inhibitor, RANK: Fc (high dose), to eliminate OC on day 14 showed no effect on bone healing (Flick et al. 2003). Similarly, op/op mice, which lack OCs due to genetic ablation of CSF-1 and exhibit an osteopetrotic (op) phenotype, showed identical soft callus removal and bone healing compared to their normal littermates (Flick et al. 2003). Moreover, in rat treated weekly with zoledronic acid, an antiresorptive medication from the bisphosphonate class, there was no delay in endochondral fracture repair (McDonald et al. 2008). The role of OCs in soft callus remodeling is still ambiguous. Further well-designed research is needed to thoroughly investigate the role of OCs in this process.

**Table 2:** Animal models for investigating the role of OCs in bone fracture healing

	**Species**	**Locus**	**OC number**	**Soft callus remodeling**	**Hard callus remodeling**	**Callus size**	**Bone healing**	**Reference**
**Genetic mouse model**								
RANKL KO	Mice	Tibia	↓	↓	↓	↑	↓	Flick et al. 2003
CTSK KO	Mice	Femur	↑	↑	↑	↓	↑	Gentile et al. 2014
PDK1 KO	Mice	Tibia	↓	↓	↓	↑	↓	Xiao et al. 2020
OPG KO	Mice	Tibia	↑	↑	N/A	N/A	↑	Ota et al. 2009
Op/op	Mice	Tibia	↓	→	N/A	N/A	→	Flick et al. 2003
**Pharmacological agent**								
RANK:Fc(high dose)	Mice	Tibia	↓	↓	→	↑	→	Flick et al. 2003
Zoledronic acid	Rats	Femur	↓	→	↓	N/A	↓	McDonald et al. 2008
Human OPG	Rats	Tibia	↓	N/A	↓	N/A	↓	Ulrich-Vinther and Andreassen 2005
Odanacatib	Rabbits	Ulnar	↑	↑	↑	N/A	↑	Pennypacker et al. 2016
Clodronate liposome	Mice	Femur	↓	↓	N/A	N/A	↓	Lin and O'Connor 2017

*Abbreviations*: *RANKL* receptor activator of NF-κB ligand, *CTSK* Cathepsin K, *PDK1* serine/threonine kinase 3‑phosphoinositide‑dependent protein kinase 1, *OPG* osteoprotegerin, *op/op* colony-stimulating factor1(CSF-1)-less osteopetrotic, *KO* knock out

In hard callus, evidence from the medaka fin ray fracture model indicates the presence of two types of OCs in hard callus during bone healing. In the early stages of fracture, smaller OCs with low TRAP activity are found at the edges of the bone fragments. In contrast, larger OCs with higher TRAP activity appear later on the inner surface of the callus (Takeyama et al. 2014). In this model, the smaller OCs facilitate fracture healing by debriding the broken bone fragments, while the larger OCs participate in callus remodeling to restore the original bone dimensions (Takeyama et al. 2014). Pharmaceutical intervention with zoledronic acid to suppress OC activity delays hard callus remodeling (McDonald et al. 2008). Similarly, in mice treated with human OPG, which significantly reduces OC numbers in tibial fractures, hard callus remodeling was greatly delayed (Table 2). This indicates that the transformation of the sizable woven bone callus into a compact lamellar structure heavily relies on OC activity (Ulrich-Vinther and Andreassen 2005).

Further evidence can be gathered from models with specific gene depletion in OCs (Table 2). Targeting serine/threonine kinase 3-phosphoinositide-dependent protein kinase 1 (PDK1) in OCs resulted in impaired OC formation and bone resorption. In a tibial fracture mouse model, the specific deletion of the PDK1 gene in OCs led to the development of a large soft callus and immature woven bone, suggesting a defective remodeling process of both soft and hard callus (Xiao et al. 2020). Conversely, genetical deletion of OPG (Ota et al. 2009) can lead to increased OC formation and accelerate cartilage resorption, which promotes early bone healing.

The precise role of OCs in fracture healing remains unclear and need more exploring. Nevertheless, based on the current evidence, OCs are critical cells and exerting its influence throughout the bone healing process.

### Osteoclasts interact with immune response after implanting biomaterials

When introducing (bio)materials or grafts into the biological environment for bone regenerative purposes, a series of immune responses promptly emerges, including acute inflammation, chronic inflammation, and foreign body reaction (Lee et al. 2019). These reactions are integral parts of the immune response involving innate immunity, with potential involvement of adaptive immune responses as well. In such cases, innate immune cells (such as macrophages, natural killer cells, etc.) and adaptive immune cells (such as T cells and B cells), along with inflammatory mediators (such as interleukins) and the complement system, actively participate (Lee et al. 2019).

The precise mechanisms underlying osteoclastogenesis and the role that OCs play through immune responses are complicated, given that OCs share regulatory molecules, such as cytokines, transcription factors, chemokines, receptors, and hormones, with various cell types (Takayanagi 2007). When implanting osteoinductive materials (i.e., inducing de novo bone formation) in mice, the immune response is triggered immediately. From a more macroscopic perspective, M0 macrophages initially polarize toward a pro-inflammatory M1 phenotype, subsequently transition to an anti-inflammatory M2 state (Guo et al. 2021). Although both M1 (Feng et al. 2023) and M2 (Dou et al. 2018b) macrophages have been reported to have the capacity to fuse into OCs. In the context of implanting osteoinductive materials, OCs emerge primarily from the fusion of M2 (Guo et al. 2021; Nie et al. 2023). This initiates the process of 'material remodeling', where they resorb the (bio)materials or grafts and release factors that promote the osteogenic differentiation of osteoblastic cell lines. It is worth noting that the fusion of M2 cells not always results in multinucleated OCs, they can also become FBGCs. Single macrophages are capable of phagocytosing particles up to 5 µm in size (Edwards et al. 1997). However, if the particle size exceeds this limit, the cells undergo fusion to form FBGCs. Studies have shown that FBGCs are capable of resorbing hydroxyapatite (HA) similar to OCs (ten Harkel et al. 2015). Herein, the attention must be paid to the interpretation of multinucleated cells on the surface of the implanted biomaterials.

### The potential role of osteoclasts in osteoinductive bone substitutes

So far, in surgical approaches of bone regeneration and augmentation autografts still represent the “gold standard’. Other types of bone substitute inferior regarding to the clinical performance (Schmidt 2021).

Calcium phosphate ceramics (CaPs) with specific surface properties have shown osteoinductive capacity and can give rise to bone formation in non-osseous locations, emerging as a promising alternative for autografts (Akiyama et al. 2011; Davison et al. 2014b; Gamblin et al. 2014; Guo et al. 2021; Kondo et al. 2006; Zhang et al. 2014). Interestingly, the osteoinductive effects triggered by these CaPs appear to have a noteworthy connection with OCs (Guo et al. 2021). However, all the evidence presented here relates to species other than humans. Stimulating osteoclastogenesis on the osteoinductive CaPs substrate in vitro, a significant population of active OCs was generated, in contrast to the non-osteoinductive CaPs, which yield limited osteoclastogenesis, the fusion of OCs were attenuated, and the OCs did not possess resorption ability (Davison et al. 2014b). In later animal studies, these two types of CaPs ceramics were implanted subcutaneously into mice and intramuscularly into dogs, respectively. The osteoinductive CaPs showed a prominent abundance of OCs alongside evident bone formation, while the control CaPs exhibited a limited number of OCs around the materials and no ectopic bone formation (Guo et al. 2021; Zhang et al. 2014). Similarly, to investigate the chronological histology of osteoinduction, β-tricalcium phosphate (β-TCP) was implanted into the dorsal muscle pouches of dogs. It was observed that a substantial population of active OCs, rather than foreign body giant cells, preceded bone formation in the peripheral material zone (Kondo et al. 2006). Subsequent studies using CaPs materials also supported these findings (Gamblin et al. 2014; Guo et al. 2021).

It is intriguing to note that in these material-induced bone formation, the appearance of bone-resorbing OCs precedes the process of new bone formation. To investigate the sequence of cellular events in CaP-initiated osteogenesis process, mice were sacrificed at various time points to identify the different cell types involved. It was reported that M0 macrophages initially polarize to the M1 phenotype and subsequently transition to an M2 state before osteoclastogenesis occurs. OCs appear earlier than bone formation and are present throughout the bone formation process (Guo et al. 2021). This phenomenon prompted a deeper exploration into the underlying mechanisms connecting OCs and ectopic bone formation on CaPs. The study employed interventions, including the use of liposomal clodronate (Davison et al. 2014a; Guo et al. 2021) or monoclonal anti-RANKL antibody (Gamblin et al. 2014; Guo et al. 2021), to suppress osteoclastogenesis following subcutaneous implantation of CaPs. The authors observed a significant inhibition in material-induced bone formation, highlighting an indispensable role of OCs in ectopic bone formation.

One of the fascinating aspects of osteoinduction by biomaterials is its strong dependence on the species of the animal. In larger animals like dogs, sheep, pigs, and primates (Le Nihouannen et al. 2005; Ripamonti 1996; Ripamonti et al. 1993; Yamasaki and Sakai 1992; Yang et al. 1996), certain biomaterials can induce bone formation within muscle tissue, even in the absence of osteogenic factors. However, in smaller animals such as rabbits, rats, and mice (Yang et al. 1996; Yuan et al. 2006), this osteoinductive effect is significantly reduced. To find a reliable mouse model for better understanding the mechanism of osteoinduction, researchers screened 11 inbred mouse strains for their responsiveness to subcutaneous implantation of osteoinductive TCP. Bone formation was observed in only two strains—FVB and 129S2—with FVB mice showing consistent bone formation in all individuals tested. The authors suggested that this variation in ectopic bone formation is likely linked to genetic differences among species and strains (Barradas et al. 2012). Further comparisons with subcutaneous implantation of osteoinductive CaPs in dogs and rats revealed distinct outcomes. In dogs, substantial ectopic bone formation was accompanied by a significant presence of OC-like cells, while in rats, bone formation was limited, and few OC-like cells were observed (Akiyama et al. 2011). These findings imply that the presence of OCs could be a key factor in material-induced osteoinduction.

Furthermore, the crucial role of OCs is not only observed based on CaPs, but also on other materials (Chen et al. 2017). In our recent work, subcutaneously implanted callus-mimetic constructs, generated by inducing chondrogenic differentiation of MSCs with a hypertrophic signature, were successfully remodeled into bone tissues in rats. Our unpublished data indicates that OC presence and TRAP signal, observed during the first two weeks post-implantation, appear to be positively related to the bone regeneration outcome of the different types of constructs. Other examples are loading bone morphogenetic protein-2 (BMP-2) on electrospun polymeric scaffolds and devitalized bovine bone granules successfully induced ectopic bone formation, accompanied by a substantial presence of OCs exhibiting a vigorous TRAP signal surrounding the construct (Husch et al. 2023). Yin and co-workers also discovered that the presence of OCs preceded osteogenesis process on nanoporous anodic alumina. Notably, the nanopore structure with a size of 200 nm exhibited a significant inhibitory effect on osteoclastic activity, resulting in the most unfavorable outcomes of osteogenesis (Chen et al. 2017). Elaborating all this evidence, it seems that the active bone-resorbing OCs are the prerequisite of material-induced bone formation, and the presence of active bone-resorbing OCs is the key of osteoinductive capacity.

In previous work, we subcutaneously implanted human macrophage- and OC-based constructs into nude mice. The results showed that these constructs failed to induce ectopic bone formation (Husch et al. 2023). The potential failing reason could be the low number of OCs loaded on the biomaterials, preventing robust resorption activities and sufficient anabolic factor release. In parallel, non-osteoinductive CaPs failed to induce bone formation also featured in restricted osteoclastogenesis with limited non-resorbing OCs formed on the surface (Zhang et al. 2014). Both limited OC numbers and resorption inability likely co-contributed to the insufficient OC-derived anabolic signal release, leading to unsuccessful bone formation. Whether the abundance of OCs with robust resorption activity is the key factor in inducing osteoinduction in material-induced bone formation, and how OCs contribute to osteoinductive capacity, requires further investigation through well-designed studies.

## The role of osteoclasts in clinically significant and prevalent bone diseases

OCs are central to the pathophysiology of several clinically significant bone diseases, including osteoporosis, osteoarthritis, and cancer-related bone remodeling (Thudium et al. 2014; Walsh and Gravallese 2004). In osteoporosis, excessive OC activity results in the loss of bone mass and structural integrity, increasing the risk of fractures (Thudium et al. 2014). In osteoarthritis, increased OC resorption activity in the subchondral bone leads to bone marrow lesions, altered joint mechanics, and cartilage breakdown (Walsh and Gravallese 2004). In cancer, particularly in bone metastases, OCs are key players in the vicious cycle of bone destruction (Gu et al. 2024). Tumor cells secrete factors such as parathyroid hormone-related peptide (PTHrP) (Guise et al. 1996), TNF-α (Li et al. 2021), IL-1 (Cozzolino et al. 1989), IL-3 (Lee et al. 2004), and IL-6 (Kawano et al. 1988) that stimulate OC formation and activation, leading to bone resorption and paving the way for metastases with osteolytic activity. In turn, OCs directly secrete factors such as PDGF (Xie et al. 2014) and BMPs (Garimella et al. 2008), or indirectly release factors from the bone matrix, such as VEGF (Cackowski et al. 2010), TGF-β (Oursler 1994), and calcium ions (Gu et al. 2024), which further fuel tumor growth. In these pathological conditions, OCs, as the primary bone-resorbing cells, become dysregulated, and their aggressive resorption activity directly contributes to the development and progression of these bone diseases. Therapies targeting OCs, such as systemic treatment with bisphosphonates (Zielińska et al. 2021), denosumab (Gnant et al. 2019) or RANKL inhibitors  (Chen et al. 2015), are important for reducing bone destruction, which in turn relieves pain and slows disease progression.

## Therapeutic strategies targeting osteoclasts in bone disease

Therapeutic interventions targeting OCs for bone diseases is an emerging area of research. For decades, OCs have been the focus of treatments for bone conditions such as osteopetrosis, osteoporosis, osteoarthritis, and bone fracture/defect healing. In the context of cancer-related bone metastasis, OC-targeted therapies have emerged over the past two decades as valuable additions to the range of existing cancer treatments. Key strategies include the use of small molecules, gene-editing technologies such as CRISPR/Cas9, and strategies based on EVs. These emerging technologies represent significant advancements in the field of OC-targeted therapies.

### Small molecules and monoclonal antibodies

Bisphosphonates and anti-RANKL antibody like denosumab are already widely used clinically for bone disease (Table 3). Bisphosphonates reduce bone resorption by promoting OC apoptosis. Consequently, bisphosphonates are widely used in treating osteoporosis by inhibiting bone resorption to achieve net bone mass gain (Reid and Billington 2022). Further, multiple methods of loading bisphosphonates onto scaffolds, e.g. via immersion (Faucheux et al. 2009), coating (Gao et al. 2009), mixing (Shi et al. 2009), and binding (Moon et al. 2011) have been extensively explored to enhance bone regeneration. In addition to their role in bone regeneration, bisphosphonates have been widely studied for their efficacy and safety in treating bone metastases from breast cancer. For example, a clinical trial with zoledronic acid, one of the most potent bisphosphonates, demonstrated a 39% reduction in the skeletal-related events (SREs) compared to placebo. Furthermore, the percentage of patients experiencing at least one SRE was reduced by 20%, the time to the first SRE was delayed, and the overall risk of SREs decreased by 41% (Kohno et al. 2005). Anti-RANKL antibody denosumab works by inhibiting the activity of RANKL to block the formation and activity of OCs. It has similar effects as bisphosphonates in treating osteoporosis (Bone et al. 2017) and cancer-related bone disease (Li et al. 2023).

**Table 3:** Small molecules and anti-body targeting OCs for bone-related disease

Small molecule/ antibody	Animal	Sexual	Time	Effect on OCs	Disease	Reference
**Bisphosphonates**						
Zoledronic acid	Human	N/A	1 year	Inhibition	breast cancer bone metastases	Kohno et al. 2005
**Anti-RANKL antibody**						
denosumab	Human	N/A	4 months	Inhibition	Solid tumors bone Metastases	Li et al. 2023
denosumab	Human	Female	10 years	Inhibition	Postmenopausal osteoporosis	Bone et al. 2017
**CTSK inhibitors**						
Odanacatib	Human	Female	5 years	Only inhibit OC resorption activity	Postmenopausal osteoporosis	McClung et al. 2019
ONO-5334	Mice	N/A	16 months	Only inhibit OC resorption activity	Osteoporosis	Yamada et al. 2016
**Src inhibitors**						
Dasatinib	Human	N/A	6 months	Inhibition	Breast cancer bone metastasis	Mitri et al. 2016
Saracatinib	Mice	N/A	4 months	Inhibition	Prostate cancer bone Metastasis	Yang et al. 2009
Bosutinib	Mice	Famale	5 weeks	Inhibition	Breast cancer bone metastasis	Jallal et al. 2007

Several CTSK inhibitors are currently in clinical development. The key distinction between CTSK inhibitors and bisphosphonates or anti-RANKL antibodies lies in their mechanism of action. CTSK inhibitors specifically target the CTSK enzyme to reduce bone resorption, while preserving the anabolic effects of OCs. In contrast, bisphosphonates and anti-RANKL antibodies reduce both the number and activity of OCs, leading to a more generalized inhibition of bone resorption and anabolic function from OCs. Odanacatib (ODN), a highly selective CTSK inhibitor, showed promise in clinical trials for osteoporosis (Eisman et al. 2011; McClung et al. 2019). ODN reduced bone resorption by inhibiting CTSK, while only transient inhibition of bone formation (Eisman et al. 2011). In a clinical trial, ODN significantly reduced the risk of fractures. However, its development was discontinued due to an increased risk of cardiovascular events (i.e. stroke) in postmenopausal women with osteoporosis (McClung et al. 2019). ONO-5334, another CTSK inhibitor, was evaluated for its effects in ovariectomized (OVX) cynomolgus monkeys, which exhibit an osteoporosis-like phenotype (Ochi et al. 2014; Yamada et al. 2016). In an 8-month treatment study, ONO-5334 significantly increased cortical bone mineral density (BMD) and improved bone mechanical strength. Notably, at a dose of 30 mg/kg, ONO-5334 did not suppress periosteal, osteonal, or endocortical bone formation rates (BFR). These findings suggest that ONO-5334 holds therapeutic potential for osteoporosis treatment (Ochi et al. 2014). In a subsequent 16-month study, ONO-5334 further increased cortical BMD, cortical area, and cortical thickness compared to control groups. Additionally, unlike alendronate treatment, ONO-5334 increased OC surface area and serum TRAP5b activity, underscoring the differences in the mechanism of action (Yamada et al. 2016).

Src plays a multifaceted role in regulating cell proliferation, survival, adhesion, migration, invasion, metastasis, and angiogenesis (Yamada et al. 2016). Mice with Src deficiency develop severe osteopetrosis due to impaired OC function (Li et al. 2024). Additionally, when cancer cells are injected into Src knock-out mice, these animals are protected from tumor-associated bone destruction, as Src-deficient OCs are unable to resorb bone (Bakewell et al. 2003). As a result, Src tyrosine kinase inhibitors show potential for treating OC-related bone diseases. However, three Src inhibitors—dasatinib (Mitri et al. 2016), saracatinib (Yang et al. 2009), and bosutinib (Jallal et al. 2007)—have undergone clinical trials in cancer patients with bone metastases. To date, the clinical outcomes in solid tumors and bone metastases have been disappointing.

The development of small molecules and antibodies targeting OCs for bone-related diseases still faces significant challenges. Several drugs based on different mechanisms have been developed, including strontium ranelate (Miranda et al. 2020), teriparatide(Parathyroid hormone related protein, PTHrP) (Black et al. 2003), and everolimus(mTOR inhibitors) (Jeong et al. 2021). However, no ideal drug has yet been identified.

### Gene-editing technologies

Gene-editing technologies, particularly CRISPR-Cas9, have opened new avenues for the treatment of bone diseases. Traditional treatments like bisphosphonates, RANKL inhibitors, and cathepsin K inhibitors aim to reduce OC activity but often come with side effects or limited efficacy. Gene-editing technologies offer a more precise approach, leading to more targeted and effective treatments.

*Engulfment And Cell Motility 1* (*ELMO1*) gene was identified for promoting enhanced OC activity. Deletion of ELMO1 in mice reduces bone loss across four in vivo models: osteoprotegerin deficiency, ovariectomy, and two types of inflammatory arthritis. Using CRISPR/Cas9 to genetically delete the Elmo1 gene in Hoxb8 macrophages (OCPs) leads to functional defects in OCs. Based on this, a 3D structure-based ELMO1 inhibitory peptide was designed and produced, which reduced bone resorption in wild-type OCs (Arandjelovic et al. 2021). This CRISPR/Cas9 gene editing technique provides a powerful tool for investigating the roles of specific genes and holds potential for developing molecular targets for the treatment of bone diseases. However, only one study has reported utilizing the CRISPR technique to target OC gene for the treatment of bone diseases by manipulating OC activity. More research and attention should be directed toward this field.

Interfering with key OC protein expression through RNA-based approaches holds significant promise. For example, transfecting pre-OCs with siRNA to silence DC-STAMP effectively inhibits their fusion and subsequent OC formation. This not only reduces bone resorption but also promotes vascularization and bone formation via increased PDGF-BB secretion (Dou et al. 2018a; Zhang et al. 2023b). Similarly, gene-editing strategies using microRNA (miRNA) to suppress critical OC genes are also being explored. Adeno-associated vectors (AAV), widely used for gene therapy, remain a reliable and efficient delivery system for both CRISPR and miRNA-based interventions (Li and Samulski 2020). In one study, the recombinant adeno-associated virus serotype 9 (rAAV9) was employed to deliver an artificial miRNA designed to silence the expression of a crucial OC regulator, *CTSK* (rAAV9.amiR-ctsk), aiming to prevent bone loss in osteoporosis. Additionally, a bone-targeting peptide motif, either (Asp)14 or (AspSerSer)6, was grafted onto the virus, ensuring bone-specific targeting. This bone-targeted rAAV9-mediated silencing of *CTSK* and effectively inhibited OC-mediated bone resorption, presenting a promising strategy for the treatment of osteoporosis (Yang et al. 2020). Similarly, AAV-mediated inhibition of miR-214-3p or overexpression of miR-34a-5p successfully reversed bone loss in mouse models of postmenopausal and senile osteoporosis by increasing OB-mediated bone formation and decreasing OC-mediated bone resorption (John et al. 2022). Moreover, miR-124 (Nakamachi et al. 2016) and miR-7b (Dou et al. 2018a) have also been explored as miRNA inhibitors of osteoclastogenesis for the treatment of osteoporosis (Table 4).

**Table 4:** MicroRNA targeting OCs for the treatment of the bone disease

MicroRNA	Animal model	Time	Delivery method	Effect on OCs	Reference
amiR-ctsk	OVX mice	2 months	Adeno-associated vectors	Only inhibit OC resorption activity	Yang et al. 2020
miR-34a-5p	OVX mice	2 months	Adeno-associated vectors	Inhibit OC formation	John et al. 2022
miR-3470b	Osteolysis model mice	2 weeks	Exosomes	Inhibit OC formation	Pan et al. 2023
miR-124	OVX mice	18 days	Injection	Inhibit OC formation	Nakamachi et al. 2016
miR-7b	OVX mice	1 month	Graphene-Based complex	Inhibit OC fuse to increase pre-OC number	Dou et al. 2018a

Gene-editing tools offer significant advantages in treating congenital genetic diseases. Mutations in the *T cell immune regulator 1* (*TCIRG1*) gene, which impair OC resorptive activity, are responsible for autosomal recessive osteopetrosis. Transfecting induced pluripotent stem cells (iPSCs) from *oc/oc* mice, which carry a deletion in the *Tcirg1* gene and closely mimic the clinical features of human osteopetrosis, with a Bacterial Artificial Chromosome (BAC) containing the full-length *Tcirg1* gene. These gene-corrected iPSC-derived myeloid cells can then differentiate into bone-resorbing OCs, offering a potential treatment for osteopetrosis. Currently, bone marrow transplantation is the only available treatment, but it is limited by the need for matched donors. In contrast, gene-editing strategies in this case using iPSCs provide an unlimited source of autologous cells, representing a promising alternative (Neri et al. 2015).

### Extracellular vesicles

EVs are membrane-derived vesicles capable of transporting cargo to both neighboring and distant target cells (S et al. 2013). There are three main subtypes: exosomes, microvesicles, and apoptotic bodies (S et al. 2013). Among these, exosomes hold the greatest promise for targeted cargo delivery, as they can be engineered to transport bioactive molecules, such as exogenous genes (Pan et al. 2023) and proteins (Faqeer et al. 2023).

In our recent study, we collected OC-derived EVs through differential centrifugation with certain modifications. SPP1 was identified as the primary osteogenesis-related cargo in these OC-derived EVs. Using these EVs for bone defect treatment significantly enhanced bone regeneration, as indicated by increased bone formation rate and volume (Faqeer et al. 2023). Another study demonstrated that exosomes derived from TNF-α preconditioned gingival MSCs have enhanced CD73 expression, inducing anti-inflammatory M2 macrophage polarization. Local injection of these exosomes significantly reduced periodontal bone resorption and decreased the number of TRAP-positive OCs (Nakao et al. 2021).

Moreover, engineered exosomes offer greater potential for multifunctionality. A recent study demonstrated that exosomes with low levels of miR-3470b, derived from macrophages, could induce osteolysis in wear particle-induced aseptic prosthesis loosening. However, by employing an engineering strategy to enrich these exosomes with miR-3470b, inhibition of OC formation was observed in vitro. Furthermore, administering the engineered miR-3470b-enriched exosomes to an osteolysis model reduced bone porosity and increased bone volume. These findings suggest that engineering exosomes with enriched miR-3470b could be a promising strategy for targeting bone resorption-related diseases (Pan et al. 2023) (Table 4). In another study, modifying MSC-derived exosomes with a bone-targeting peptide enabled them to specifically target bone tissue. These exosomes, loaded with siRNA targeting the Shn3 gene via electroporation, silenced the osteoblastic Shn3 gene, enhancing osteogenic differentiation, reducing autologous RANKL expression, and inhibiting OC formation. Additionally, Shn3 gene silencing increased SLIT3 production, promoting vascularization, particularly the formation of type H vessels. As a result, these bone-targeted, siShn3-loaded exosomes simultaneously address excessive bone resorption, insufficient bone formation, and inadequate vascularization—three key factors in the pathogenesis of osteoporosis (Cui et al. 2022).

Another promising aspect is the potential use of OC-derived EVs in biomarker discovery. These vesicles reflect the physiological state of the cells from which they are released, for which they can serve as indicators of OC activity and bone disease progression. Elevated levels of miR-21 in exosomes have been proposed as a biomarker for clinical diagnosis and treatment of breast cancer bone metastasis (Yuan et al. 2021).

## Conclusions and perspectives

The present review gathered the current evidence to depict the process of OC formation, from origin to formation via fusion, and the role of OCs in bone formation and regeneration. The time of occurrence and the source of origin of OCs at different development stage currently gives different insights, as compared to previous understanding (Jacome-Galarza et al. 2019; Yahara et al. 2020). However, OCs remain enigmatic, as their biology is not yet fully understood. For instance, achieving pure OC cultures in vitro under normal conditions has proven challenging, with a significant number of unfused precursor cells consistently observed around the multinucleated OCs (Husch et al. 2021). Our team has made efforts in this area, successfully obtaining a pure OC population (Husch et al. 2024). This, however, still requires specific techniques, such as using microgels to microencapsulate OCPs to facilitate OC formation. Furthermore, 100% OC formation within hollow microgels has not been realized, and cell sorting based on specific markers is still necessary to isolate pure OCs (Husch et al. 2024). A comprehensive understanding of the process by which OCPs fuse into OCs is crucial. Current evidence suggests that OC fusion is largely heterogeneous, with 62% of fusion events occurring between mobile and immobile partners, and nearly 70% of multinucleated OCs fusing with mononucleated OCPs (Søe et al. 2015). Only 2.4% of OCPs act as initiators of the fusion process (Levaot et al. 2015). Identifying and characterizing these fusion-initiating OCPs would be invaluable, as it may help pinpoint the specific OCP population responsible for initiating the fusion process. If it becomes possible to pre-sort OCP initiators for pure culturing, significantly higher OC formation rates could be achieved, especially given that OCs represent only 3.8% ± 0.8% of the cell population in conventional 2D cultures (Husch et al. 2024).

Identifying OCs in vivo can be challenging, as OCs are not the only cells that exhibit a multinucleated structure (Ahmadzadeh et al. 2022). Therefore, we summarized the most commonly used cellular characteristics of OCs to provide information for identifying OCs in heterogeneous cell populations in both in vivo and in vitro situations. Due to the non-availability of a single unique marker displayed in OCs, we recommend combining cellular markers (e.g., TRAP, integrin β3, Vpp3) with cellular structures (e.g. multinucleation) as the most reliable identification method of OCs. Additionally, if available, the substrate of the cells (e.g. bone) should be considered in order to confirm the identity of OCs.

OCs are primarily known for their bone resorption ability, but their roles beyond resorption are often overlooked. In this review, we highlight evidence of OCs' roles in bone marrow cavity formation, angiogenesis, and bone remodeling to shed light on their contribution to physiological bone formation. In Sect. 5.3, we emphasize how OCs first appear at resorption sites and later initiate osteogenesis process during bone remodeling. Interestingly, in osteoinductive material-induced bone formation, OCs also appear before bone formation begins (Guo et al. 2021), and depletion of these OCs significantly impairs subsequent bone formation (Guo et al. 2021). Whether the role of OCs in physiological bone remodeling mirrors their function in 'material remodeling' remains unclear. Furthermore, it is still uncertain whether OCs contribute directly to osteoinduction. The rationale behind this speculation includes: (i) OCs initiate osteogenesis process in physiological bone remodeling and may play a similar role in biomaterial-induced bone formation; (ii) OCs secrete or release factors such as TGF-β1 and SPP1, which induce the migration of MSCs or OBs to bone surfaces for subsequent bone formation; (iii) OCs promote bone formation by inducing angiogenesis, a prerequisite for bone formation that supports the recruitment of OBs and the supply of nutrients. Further investigation is needed to address these questions.

To date, most strategies for stimulating bone formation in regenerative therapies focus on increasing the numbers and activity of OBs and their precursors (Anjum et al. 2023; Hu et al. 2023; Luo et al. 2023; Mounier et al. 2020). However, no bone substitutes have yet achieved ideal bone formation or regeneration in terms of both rate and volume. This raises the question of whether we are focusing on the wrong cell types in promoting bone formation. As discussed in this review, OCs possess anabolic capacity in bone formation. Therefore, strategies aimed at reducing OCs to enhance bone formation may fall short of realizing the full potential of bone regeneration. Shifting focus towards harnessing OC activity to stimulate bone formation could be more promising in the future.

The major role of OCs in prevalent bone diseases like osteoporosis, osteoarthritis, and cancer-related bone metastasis lies in their bone-resorption ability. Several therapeutic strategies have been developed to address this clinical issue (Bone et al. 2017; Kohno et al. 2005). Small molecules or antibodies are the most extensively studied drugs, many of which have undergone multiple clinical trials and are already commercially available and used in clinical practice. However, none of these treatments have demonstrated ideal effects in terms of minimal side effects or optimal outcomes. As a result, there has been growing interest in approaching these diseases from a genetic perspective. Gene-editing tools have the potential to alter or correct gene expression, thereby modifying the production of functional proteins to permanently treat bone-related diseases, rather than relying on continuous drug intake. CRISPR/Cas9 has emerged as a powerful tool in this field, but only one study so far has specifically targeted the OC gene (Arandjelovic et al. 2021). On the other hand, various RNA molecules (siRNA and miRNA) have been widely used in this area. For delivering these RNA molecules to modify gene expression, EVs—especially exosomes—show great promise as they are stable, small, and capable of specific targeting due to their surface proteins (Nakao et al. 2021; Pan et al. 2023). Engineered cargos, such as RNA, can be loaded into exosomes, and these exosomes can also be modified to target specific locations like bone (Cui et al. 2022), further enhancing their specificity. However, the safety of using EV-based therapeutic treatments requires further investigation. Utilizing genetic approaches combined with EVs as delivery vehicles holds significant potential for treating bone diseases; However, no mature EV-based therapies for bone-related diseases have been developed to date. The feasibility of large-scale production or economically viable options remains uncertain, leaving many scientific challenges in this field to be resolved.

## Data Availability

Not applicable.

## Abbreviations

OCs: Osteoclasts
OCPs: Osteoclast precursors
OBs: Osteoblasts
MSCs: Mesenchymal stem cells
EMPs: Erythromyeloid progenitors
HSCs: Hematopoietic stem cells
M-CSF: Macrophage colony-stimulating factor
RANKL: Receptor activator of nuclear factor kappa-B ligand
TRAP: Tartrate-resistant acidic phosphatase
CSF1R: Colony-stimulating factor 1 receptor
AGM: Aorta-gonad-mesonephros
MPPs: Multipotent progenitors
CLPs: Common lymphoid progenitors
CMPs: Common myeloid progenitors
MEPs: Megakaryocyte–erythrocyte progenitors
GMPs: Granulocyte-monocyte progenitors
DCs: Dendritic cells
DC-STAMP: Dendritic cell-specific transmembrane protein
ASCT2: Amino acid transporter 2
OSCAR: Osteoclast-associated receptor
CTR: Calcitonin receptor
CAII: Carbonic anhydrase II
CTSK: Cathepsin K
MMP: Matrix metalloproteinase
V-ATPase: Vacuolar ATPase
Vpp3: Vacuolar-type proton pump-3
OPG: Osteoprotegerin
ECs: Endothelial cells
ANG: Angiogenin
PDGF-BB: Platelet-derived growth factor-BB
VEGFs: Vascular endothelial growth factors
RCs: Reversal cells
TGF-β: Transforming growth factor-β
IGF1: Insulin-like growth factor 1
EFNB2: Ephrin B2
SEMA: Semaphorin
CT-1: Cardiotrophin-1
S1P: Sphingosine 1 Phosphate
CTHRC1: Collagen Triple Helix Repeat Containing 1
C3: Complement Component C
EVs: Extracellular vesicles
SPP1: Secreted phosphoprotein 1
SMAD3: Smad family member 3
PDK1: Serine/threonine kinase 3‑ phosphoinositide-dependent protein kinase 1
Op/op: Colony-stimulating factor1(CSF-1)-less osteopetrotic
TRPV1: Transient receptor potential vanilloid
IL-6: Interleukin-6
STZ: Streptozotocin
KO: Knock out
NFATc1: Nuclear factor of activated T-cells cytoplasmic 1
FBGCs: Foreign body giant cells
CaPs: Calcium phosphate ceramics
β-TCP: β-tricalcium phosphate
BMP: Bone morphogenetic protein
